# Novel Anticancer Strategies

**DOI:** 10.3390/pharmaceutics13020275

**Published:** 2021-02-18

**Authors:** Hassan Bousbaa

**Affiliations:** CESPU, Instituto de Investigação e Formação Avançada Em Ciências e Tecnologias da Saúde, Instituto Universitário de Ciências da Saúde, 4585-116 Gandra PRD, Portugal; hassan.bousbaa@iucs.cespu.pt

Cancer incidence and mortality continue to increase rapidly worldwide. Owing to the dynamic, rapid, and adaptive nature of cancer progression, side effects and resistance associated with the existing therapies provide continuous and challenging exercises for searching for additional drugs and drug delivery strategies with the goal of offering more effective therapeutic options. Therefore, novel therapeutic strategies are constantly needed in order to overcome the drawbacks associated with the strategies in use in the clinic. This Special Issue (https://www.mdpi.com/journal/pharmaceutics/special_issues/novel_anticancer (accessed on 11 February 2021)) is dedicated to innovative research on the development and validation of novel anticancer approaches, hopefully with relevant clinical value in the near future. In this sense, we have received interesting contributions, in the form of original works and reviews, that cover innovative drug delivery systems, improvement of the efficacy of approved anticancer agents, and validation of new anticancer drugs.

Nanoparticles are 1 to 100 nm sized materials and are grouped into different classes according to their properties, shapes, or sizes. They have a wide range of applications in modern medicine, namely, as carriers for drug and gene delivery into tumors. Due to their unique proprieties, mesoporous silica nanoparticles (MSNs) have deserved increasing interest in nanoparticle-mediated drug delivery research. MSNs are chemically stable with good biocompatibility, which, when adequately tailored, can provide large surface area and pore volume for high loading capacity, as well as selective and controlled delivery of therapeutic agents. Particularly interesting is the effective delivery potential of MSNs of poorly soluble anticancer agents, namely, prodrugs derived from different natural origins, as reviewed by Abouaitah and Lojkowski [1].

Dendrimers and polymeric micelles are other promising drug delivery systems. Dendrimers are regularly hyperbranched and mainly 3D macromolecules. They are nanosized radially symmetric molecules with a well-defined, homogeneous, and monodisperse structure. These characteristics make dendrimers a good choice for the delivery of anticancer drugs. Polymeric micelles are formed by the spontaneous arrangement of amphiphilic block copolymers in aqueous solutions. Their hydrophobic core–hydrophilic shell architecture facilitates the loading of hydrophobic drugs into the core, providing a main to improve the solubility of anticancer drugs. Alven and Aderibigbe summarize the application and outcomes of dendrimers and micelles loaded with different known anticancer agents using in vitro and in vivo models of breast cancer [2]. Jin et al. report the use of Soluplus polymeric micelles to encapsulate the veterinary anthelmintic fenbendazole (FEN), also known for its anticancer efficacy, in order to overcome its low solubility problem [3]. They observed that micellar formulation exhibited superior bioavailability compared with that of free FEN, with no severe toxicity, as revealed by in vivo toxicity assay, thereby paving the way for preclinical and clinical safety and efficacy trials on FEN-loaded Soluplus micelles.

Nanotechnology could be a promising solution to overcome delivery and resistance concerns of currently available chemotherapeutics against glioblastoma, one of the most aggressive types of cancer. Low drug solubility, blood–brain barrier penetration, and drug–target residence time are major issues in translating in vitro potency to in vivo efficacy for glioblastoma treatment. In their review, Paranthaman et al. describe the molecular basis of glioblastoma, emphasizing the role of receptor tyrosine kinases (RTKs) and small molecules under clinical trials, and provide an updated research literature and future guidelines on epidermal growth factor receptor (EGFR)-RTK inhibitors-based nanodelivery systems [4].

Deep tumor penetration of drug-loaded nanoparticles can be compromised by a dense extracellular matrix (ECM), thereby limiting their therapeutic efficacy. To overcome this issue, Choi et al. used high-intensity focused ultrasound (HIFU) technology to improve the tumor penetration of doxorubicin (DOX)-loaded glycol chitosan nanoparticles (CNPs) [5]. The treatment of ECM-rich tumor-bearing mice with HIFU resulted in an increased accumulation of DOX-CNPs at targeted tumor tissues via deep tumor penetration, through HIFU-mediated dense ECM destruction, providing a means to increase deep penetration into heterogeneous tumors with dense ECM structures. In another study, Haugse et al. analyzed the mechanism behind the use of ultrasound and microbubbles—known as sonoporation—in improving the efficacy of chemotherapy [6]. They observed that sonoporation was associated with an immediate transient activation of MAP kinases (p38, ERK1/2) and an increase in phosphorylation of ribosomal protein S6 together with dephosphorylation of 4E-BP1, a stress response resembling cellular responses to electroporation and pore-forming toxins. Their data also suggest that cells in the tumor microenvironment may be relevant for sonoporation efficacy, which could be exploited therapeutically. However, such analysis should be performed on heterotypic 3D cultures so as to recapitulate the patient tumor architecture and the heterogeneity of cell types and cell–cell interactions [7].

A natural solution for drug delivery is provided by extracellular vesicles (EVs), which are lipid-bound vesicles secreted by cells into the extracellular space, with key roles in intercellular communication. EVs include microvesicles, exosomes, and apoptotic bodies, whose content consists of lipids, nucleic acids, and proteins. Although their isolation and analysis methods still suffer from a lack of standardization, EVs have unique features that are relevant for drug delivery and are expected to overcome the inefficiency, cytotoxicity, and/or immunogenicity associated with synthetic delivery systems, as reviewed by Hernandez-Oller et al. [8]. Park et al. report the use of tumor-homing pH-sensitive EV blends made from tumor-specific EVs, extracted from two different tumor cell types, and pH-sensitive HDEA (3-(diethylamino)propylamine). These EVs were loaded with hyaluronic acid grafted with HDEA and doxorubicin (DOX, as a model antitumor drug) [9]. HDEA/DOX-anchored EVs were able to target the two different parent tumor cells owing to the EVs’ homing ability. The pH-sensitive disruption of EVs, owing to DEAP (3-(diethylamino)propylamine) molecules, promoted DOX release, resulting in the effective killing of the heterogeneous parent tumor cells. The finding highlights the potential of EV blends as effective targeted therapies for various tumor cells.

Specific addressing of tumor cells while sparing healthy tissues is currently a major desire in cancer therapy. Tumor-specific binding agents can be conjugated to an anticancer drug to guide the drug to the targeted the tumor. Dókus et al. developed peptide-based drug conjugates against pancreatic cancer cells (PANC-1) [10]. They used the SKAAKN hexapeptide, derived from the previously reported CKAAKN sequence by the substitution of Cys to Ser, in conjugates containing daunomycin (Dau). One conjugate exhibited significant tumor growth inhibition on PANC-1 tumor-bearing mice with negligible side effects, highlighting the promising potential of peptide-based drug delivery systems for pancreatic cancer treatment. Due to their rapid blood clearance, repeated administration of peptide-based therapeutic agents may be necessary. Abouzayed et al. report a successful conjugation of the gastrin-releasing peptide receptor (GRPR) antagonist RM26 and an albumin-binding domain. The conjugate retained GRPR targeting in vivo and, due to binding to albumin, resulted in an increased residence time in blood and in tumors, while retaining specificity and its antagonistic function against GRPR [11]. Although its use for radionuclide therapy is precluded due to undesirable elevated activity uptake in kidneys, the approach deserves further optimization.

Cancer recurrence arises from the incomplete eradication of tumor cells after chemo- and radiotherapy, being one of the major reasons for the failure of cancer treatment strategies. Cancer stem cells (CSCs) are believed to be one of the important drivers of cancer relapse. CSCs are characterized by self-renewal capacity and differentiation potential. Various cancers include a small population of CSCs that confer them metastasis, heterogeneity, drug and radiation resistance, and tumor relapse. In this sense, Quiroz-Reyes et al. provide a comprehensive review on conventional and novel developments in cancer therapeutics for liver, lung, and pancreatic metastasis, with a focus on CSCs as a valuable target to eradicate tumor relapse [12]. Further targeting of CSCs and cancer resistance, using EVs as natural drug delivery systems, is also reviewed by Hernandez-Oller et al. [8].

Arsenic derivatives have been shown to exert anticancer effects, namely, by inducing apoptosis, providing a new alternative to classical chemotherapeutics and radiotherapy. For instance, arsenic trioxide (As_2_O_3_, Trisenox) has been approved for the treatment of acute promyelocytic leukemia. Noh et al. report the anticancer effects of tetraarsenic hexoxide (TAO, As_4_O_6_) in a series of patient-derived xenograft (PDX) mouse models of cervical cancer [13]. They showed that TAO induced significant anticancer effect in PDXs with primary cancers, and when combined with cisplatin, PDXs with recurrent cancers were also significantly inhibited. This highlights the potential usefulness of TAO for cervical cancer treatment.

A major obstacle in translating discoveries from preclinical research (bench) into human applications for cancer therapy (bed) resides in the fact that preclinical models do not mimic the real tumor microenvironment. For instance, 2D monolayer cell cultures have reduced cell–cell contacts and lack interactions with a surrounding extracellular framework in three dimensions. In this sense, 3D tumor models, by recapitulating relevant properties of tumor microenvironment interactions, promise to bridge the gap between 2D cell culture and in vivo experiments, and advance our current understanding of cancer. Pinto et al. provide a concise and useful review of the current techniques used to prepare and analyze in vitro 3D spheroids, and discuss the significance of 3D cultures in drug resistance for the evaluation of the therapeutic efficacy of nanomedicines [7]. Using in vitro 2D and 3D spheroid models, Sicard et al. conducted a pilot study to demonstrate that antiproliferative efficacy against prostate cancer (PCa) can be achieved by encapsulating antisense oligonucleotide (ASO) into liposomes to silence TCTP [14]. Interestingly, the most promising efficacy on 3D spheroids was achieved with immunoliposomes targeting Her2, provided that incubation time was long enough, despite a low expression of Her2 in PCa cells.

Cancer immunotherapy (IT) has brought a new hope to cancer patients. The use of immune checkpoint inhibitors has led to a net improvement of survival and quality of life, when compared with standard therapies. However, its use is restricted to very limited cancer types. To extend its use to a larger number of cancers, immune checkpoint inhibitors are being combined with standard therapeutic strategies. However, due to immunomodulating features, an optimal time window to combine immune checkpoint inhibitors with other drugs needs to be defined in order to achieve maximum efficacy while controlling toxicities. Sicard et al. describe the putative biomarkers that could help define this window, with a special focus on circulating tumor DNA whose detection indicates that the STING–cGAS pathway is activated by the immune checkpoint inhibitors [15]. Still in the field of IT, Byun et al. modeled the tumor-immune interactions occurring during combined IT and ionizing irradiation therapy, and suggest that the ratio of PD-1 to PD-L1 in T cells could be considered in combination therapy [16].

In summary, persistence in developing novel anticancer strategies is inevitable to face the adaptive nature of cancer progression. New anticancer drugs are always welcome as alternatives to circumvent side effects and resistance to existing drugs. It is noteworthy that the use of preclinical models that mimic the real tumor microenvironment is important to speed up the translation of research from lab to clinic. Further efforts are also needed to maximize the efficacy of existing drugs, either by chemical modifications or by specific targeting to the desired tumor. Approaches that provide deep penetration of nancarriers into heterogeneous tumors with dense ECM structures bear tremendous potential to improve the efficacy of chemotherapy. The contributions published in this Special Issue are examples of progress towards the achievement of such objectives.
